# Association of Over-Expressed Estrogen Receptor Alpha with Development of Tamoxifen Resistant Hyperplasia and Adenocarcinomas in Genetically Engineered Mice

**DOI:** 10.4172/2161-0940.s12-001

**Published:** 2012-06-25

**Authors:** Anne M Miermont, Marina Carla Cabrera, Silvina M Frech, Rebecca E Nakles, Edgar S. Diaz-Cruz, Maddalena Tilli Shiffert, Priscilla A Furth

**Affiliations:** 1 Department of Oncology, Lombardi Comprehensive Cancer Center, Georgetown University, Washington, DC, 20007, USA; 2 Department of Biology, Georgetown University, Washington, DC, 20007, USA; 3 Department of Medicine, Lombardi Comprehensive Cancer Center, Georgetown University, Washington, DC, 20007, USA; 4 Department of Nanobiomedical Science and WCU Research Center of Nanobiomedical Science, Dankook University, Chungnam 330-714, Korea

**Keywords:** ERα, Cyclin D1, Breast cancer, Hyperplasia, Tamoxifen, Mouse models

## Abstract

**Background:**

Estrogen receptor alpha (ERα) and cyclin D1 are frequently co-expressed in human breast cancer. Some, but not all, studies link tamoxifen resistance to co-expression of cyclin D1 and ERα. In mice over-expression of either cyclin D1 or ERα in mammary epithelial cells is sufficient to induce mammary hyperplasia. Cyclin D1 over-expression in mice leads to mammary adenocarcinoma associated with activated estrogen signaling pathways. ERα over-expression in mice leads to mammary hyperplasia and cancer. Significantly, disease development in these mice is abrogated by loss of cyclin D1.

**Methods:**

Genetically engineered mouse models were used to determine whether or not ERα over-expression demonstrated cooperativity with cyclin D1 over-expression in cancer development, reaction to the chemical carcinogen DMBA, or tamoxifen response.

**Results:**

Adding ERα over-expression to cyclin D1 over-expression increased the prevalence of hyperplasia but not cancer. Single dose DMBA exposure did not increase cancer prevalence in any of the genotypes although cyclin D1 over-expressing mice demonstrated a significant increase in hyperplasia. Tamoxifen treatment was initiated at both young and older ages to test for genotype-specific differences in response. Although normal ductal structures regressed in all genotypes at both younger and older ages, tamoxifen did not significantly reduce the prevalence of either hyperplasia or cancer in any of the genotypes. All of the cancers that developed were hormone receptor positive, including those that developed on tamoxifen, and all showed expression of nuclear-localized cyclin D1. In summary, development of tamoxifen resistant hyperplasia and cancer was associated with expression of ERα and cyclin D1.

**Conclusion:**

These preclinical models will be useful to test strategies for overcoming tamoxifen resistance, perhaps by simultaneously targeting cell cycle regulatory pathways associated with cyclin D1.

## Introduction

Estrogen Receptor alpha (ERα) and cyclin D1 are frequently although not invariably co-expressed in invasive human breast cancers [1–5]. Increased levels of ERα and cyclin D1 expression are reported in precursor lesions and non-invasive breast cancers [6–9]. The two proteins share a molecular interplay. Cyclin D1 lies downstream of estrogen signaling through ERα [10–13] but cyclin D1 also lies upstream of ERα and can activate ERα driven transcription through cyclin dependent kinase (CDK) independent mechanisms [14–16]. ERα and cyclin D1 also activate the same set of genes linked to breast cancer progression [17].

Combination estrogen and progesterone hormone replacement therapy in post-menopausal women significantly increases breast cancer risk [18–20]. Cyclin D1 positively regulates progesterone receptor expression levels and this has been postulated to be a factor that contributes to the increased risk of cancer development following hormonal exposure [21]. In ERα positive breast cancer, coincident cyclin D1 expression in untreated women has been repeatedly presented, although not always, as a positive prognostic factor [1,2,4,5]. Some studies have linked expression of cyclin D1 in ERα positive breast cancers to an impaired response to tamoxifen [22–28] while others show no statistically significant effects [29,30].

Genetically engineered ERα over-expression in mammary epithelial cells in transgenic mice leads to development of ductal hyperplasia and ductal carcinoma in situ (DCIS) lesions by four months of age [31], hyperplastic alveolar nodules (HANs) by eight months of age [32], and both ERα positive and ERα negative invasive mammary cancers by 12 months of age following single-dose treatment with the chemical carcinogen 12-dimethylbenz[a]anthracene (DMBA) [33]. Cyclin D1 is found in the ductal hyperplasias, DCIS, and invasive cancers. Loss of cyclin D1 in this mouse model, referred to as Conditional Estrogen Receptor in Mammary tissue (CERM) mice, leads to a DNA damage response in response to pubertal estrogen stimulation that causes apoptotic death of the mammary epithelial cells and abrogates normal mammary gland development [34]. ERα over-expressing CERM mice express ERα at approximately two-fold higher levels than normal [31,32,35,36], comparable to what is described in human preneoplastic breast tissue [9,37]. This results in increased *Erbb2*, *Efgr*, *Pgr*, *Tnsfs11* RNA expression, and phosphorylated mitogen-activated protein kinase 3/1 (ERK1/2), mitogen-activated protein kinase 8 (JNK), signal transducer and transactivator (STAT)3, and STAT5 protein expression in normal-appearing mammary tissue [32,35]. High percentages of precancerous and cancer cells exhibit increased cyclin D1, antigen identified by monoclonal antibody (Ki67), proliferating cell nuclear antigen (PCNA), phosphorylated ERK1/2 and phosphorylated STAT5 [31–33,35].

Genetically engineered cyclin D1 over-expression targeted to the mammary epithelial cells of transgenic mice leads to development of preneoplasia in young mice followed by cancer development when mice are older than one year [21,38]. Cyclin D1 expression levels in these mice are comparable to those found in cultured human breast cancer cells [38]. Increased expression of cyclin D1-regulated genes including progesterone receptor A, cyclin A2, immediate early response 3, small stress protein 1, and tumor necrosis factor-associated factor-interacting protein starts in preneoplasia and is believed to contribute to carcinogenic transformation [17,21].

Mice over-expressing cyclin D1 were crossed with CERM mice to determine if ERα and cyclin D1 would demonstrate cooperativity *in vivo* in the development of hyperplasia and cancer. Cohorts of mice were exposed to single-dose DMBA [33,39] to test if the combination of over-expressed cyclin D1 with ERα increased susceptibility to this carcinogenic insult. The response to tamoxifen was tested at two different ages (four and ten months of age) to test if tamoxifen could cause regression of preneoplasia and cancer initiated by over-expression of ERα and cyclin D1 either alone or in combination.

## Materials and Methods

### Mouse models

Bi-transgenic *tet-op-ERα^Mouse Mammary Tumor Virus (MMTV)^* (termed Conditional Estrogen Receptor in Mammary tissue or CERM) mice carrying *tet-op-ERα* and *MMTV-rtTA* transgenes [31–36] were crossed with *MMTV-cyclin D1* (termed D1) transgenic mice [21,38] to generate female CERM (n=30), CERM/D1 (n=52) and D1 (n=42) mice on a C57Bl/6 background. C57Bl/6 wild-type (WT) mice were used as controls in the no-intervention and tamoxifen-intervention studies (n=13). Detection of the *MMTV-rtTA*, *tet-op-ERα* and *MMTV-cyclin D1* transgenes was performed from tails by polymerase chain reaction (PCR) (Transnetyx, Cordova, TN). Mice were maintained on a 12-hour light/dark cycle with a doxycycline-containing diet (Bio-Serv, Frenchtown, NJ) with water ad libitum. All genotypes demonstrated equivalent growth, activity, and fertility, and the Georgetown University Animal Care and Use Committee and Institutional Biosafety Committee approved all procedures.

### Time-course and intervention studies

A no-intervention time-course study without any treatment was performed to determine if there were any genotype-specific differences in prevalence of hyperplastic alveolar nodules (HANs) at one year of age or development of mammary cancer by one year of age in CERM (n=16), CERM/D1 (n=25) and D1 (n=20) mice. A smaller control cohort of WT (n=4) mice was followed to provide non-transgenic mammary gland whole mounts (WMs) for comparative examination with the experimental mice. An interventional time-course study following a single intragastric 1 mg dose of the chemical carcinogen 12-dimethylbenz[a]anthracene (DMBA) (D3254, Sigma) at four months of age [33,39] was performed to determine if there were any genotype-specific differences in prevalence of HANs or development of mammary cancer at one year of age in CERM (n=4), CERM/D1 (n=10) and D1 (n=9) mice following exposure to this chemical carcinogen. An interventional time-course study following tamoxifen treatment initiated at ten months of age was performed to determine if there were any genotype-specific differences in prevalence of HANs by one year of age or development of mammary cancer by one year of age in CERM (n=10), CERM/D1 (n=17) and D1 (n=13) mice. A control cohort of WT (n=9) mice was followed to provide non-transgenic mammary gland whole mounts for comparative examination with the experimental mice. An interventional time-course study following tamoxifen treatment initiated at four months of age was performed to determine if there were any genotype-specific differences in prevalence of HANs at six months of age or development of mammary cancer by six months of age in CERM (n=16), CERM/D1 (n=6) and D1 (n=4) mice. A control cohort of WT mice (n=19) was followed to provide non-transgenic mammary gland whole mounts for comparative examination with the experimental mice. Mice were implanted subcutaneously with a 25 mg 60-day constant release tamoxifen pellet (Innovative Research of America, Sarasota, FL). All mice were followed with weekly clinical examination until necropsy timepoints at age six or 12 months, or when tumor reached 1 cm^3^ or other health issues required euthanasia.

### Histology and mammary gland whole mount studies

At the time of necropsy one inguinal mammary gland was taken for mammary gland whole mount analyses and palpable tumors were isolated from surrounding mammary tissue, formalin-fixed, embedded and sectioned for histological analyses. Hematoxylin and eosin (H&E)-stained sections were used to evaluate histology of the tumors. All tumors were classified as invasive adenocarcinomas. For immunohistochemistry, paraffin-embedded mammary gland sections were deparaffinized in three successive five minute xylene incubations, and rehydrated in two successive three minute each incubation steps of 100%, 95% and 75% followed by 50% ethanol for two minutes. Endogenous peroxidase was quenched with 3% hydrogen peroxide for 10 min and sections washed twice in dH_2_O for two minutes. Antigen retrieval was performed either in a decloaker using BORG retrieval solution (Biocare Medical, Concord, CA) or by immersion at 98°C for 20 minutes in 10 mM citrate buffer (pH 6.0) with 0.05% Tween. Primary antibodies: ERα (Santa Cruz, sc-542) (1:750 1 hr RT), PgR (Santa Cruz, sc-538) (1:250 1 hr RT), cyclin D1 (Neomarkers, RM-9104, Fremont, CA) (1:50-100 overnight 4°C or 1 hr RT), Ki67 (Novacastra, NCL-Ki67-MM1, Logan, UT) (1:25-1:100 1 hr RT), and ErbB2 (Santa Cruz, sc284). Immunohistochemical staining was performed using the rabbit VectaStain or the Mouse-On-Mouse kit (Vector Labs, Burlingame, CA). Slides were exposed to biotin-conjugated anti-rabbit or anti-mouse secondary antibodies, VectaStain ABC reagent and DAB chromate (Dako, Carpinteria, CA) to detect horseradish peroxidase, counterstained with Hematoxylin (Vector Labs), dehydrated, and mounted with VectaMount or Acrymount (Vector Labs). Mammary gland whole mounts were examined under low power magnification (0.5X, 1X, 4X) to identify and count HANs. The percentage of mice with at least one HAN per mammary gland and the percentage of mice with more than one (multiple) HANs per mammary gland were calculated and recorded for each genotype at the one-year timepoint. Digital images were captured using a Nikon Eclipse E800M microscope and DMX1200 camera with the ACT-1 Version 2.7 program (Nikon Corporation, Melville, NY).

### Statistical analyses

Statistical analyses were performed with GraphPad Prism version 4 for Windows (La Jolla, CA). HAN, multiple HANs and adenocarcinoma prevalence were compared using Fisher’s Exact Test (one-tailed test). Statistical significance was reached when p ≤ 0.05.

## Results

### Addition of ERα over-expression to cyclin D1 over-expression increased prevalence of mammary hyperplasia but not adenocarcinoma

**Figure 1:**
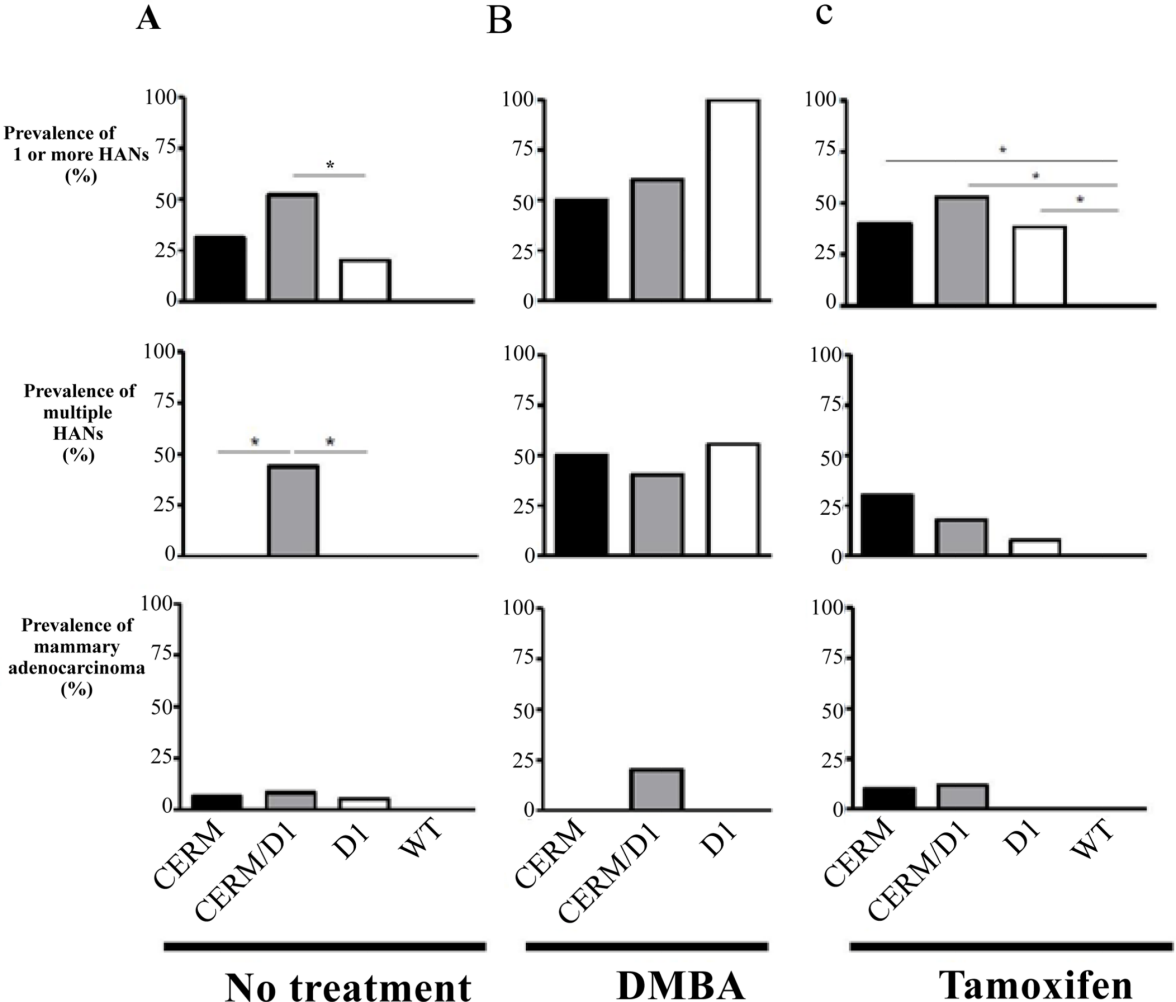
Prevalence of hyperplastic alveolar nodules (HANs) and invasive adenocarcinomas in one-year-old mice over-expressing ERα (CERM), cyclin D1 (D1), both ERα and D1 (CERM/D1) mice in no-treatment and DMBA and tamoxifen intervention groups **(A)** Bar graphs comparing the prevalence of at least one HAN, multiple HANs, and invasive cancers in one-year-old CERM, CERM/D1, D1 and WT mice that received no treatment intervention. **(B)** Bar graphs comparing the prevalence of at least one HAN, multiple HANs, and invasive cancers in one-year-old CERM, CERM/D1, and D1 mice that were exposed to a single-dose of DMBA intervention at four months of age. **(C)** Bar graphs comparing the prevalence of at least one HAN, multiple HANs, and invasive cancers in one-year-old CERM, CERM/D1, D1 and WT mice that received tamoxifen treatment intervention at ten months of age. * indicates statistically significant differences (Fisher’s exact test, one tailed, p≤0.05).

The addition of ERα over-expression to cyclin D1 over-expression in the CERM/D1 mice significantly increased the percentage of mice demonstrating one or more HANs at one year of age (Fisher’s exact, one tailed test, p≤0.05); however, it did not increase the number of mice developing mammary adenocarcinoma (Figure 1A). Notably, only mice over-expressing both ERα and cyclin D1 demonstrated multiple HANs (Figure 1A).

### DMBA exposure increased prevalence of mammary hyperplasia but not adenocarcinoma in mice over-expressing cyclin D1

Single-dose DMBA exposure at four months of age was used to investigate if this chemical carcinogen could increase the percentage of mice demonstrating HANs or adenocarcinoma at age one year in CERM, CERM/D1 or D1 mice. Only D1 mice demonstrated a significant increase in HAN prevalence compared to the mice without any treatment (Fisher’s exact, one-tailed test, p≤0.05) but none developed adenocarcinoma (Figure 1B). While adenocarcinoma development following single-dose DMBA treatment was limited to the CERM/D1 mice (Figure 1B), the percentage found was not statistically significantly different from untreated mice.

### Tamoxifen treatment at ten months of age did not reduce prevalence of mammary hyperplasia or adenocarcinoma induced by over-expression of ERα or cyclin D1

Tamoxifen treatment was initiated at ten months of age to determine if this established breast cancer preventive could reduce the percentage of mice demonstrating either HANs or adenocarcinoma in CERM, CERM/D1 or D1 mice by age one year. Notably, the percentage of mice demonstrating HANS or adenocarcinoma was not significantly different from untreated mice (Figure 1A and 1C). However, while none of the no-intervention CERM or D1 mice demonstrated multiple HANs, over 25% of the tamoxifen-treated CERM and 5% of the D1 mice exhibited more than one HAN, a significant increase from mice without any treatment (Fisher’s exact, one-tailed test, p ≤ 0.05) (Figure 1A and 1C). At the time tamoxifen treatment was initiated no mice demonstrated palpable tumors; however adenocarcinomas developed on tamoxifen treatment in CERM (n=1) and CERM/D1 (n=2) mice (Figure 1C).

### Mammary hyperplasia following tamoxifen treatment was a time-dependent acquired phenotype at ten months of age and not directly associated with either ERα or cyclin D1 transgene expression

**Figure 2:**
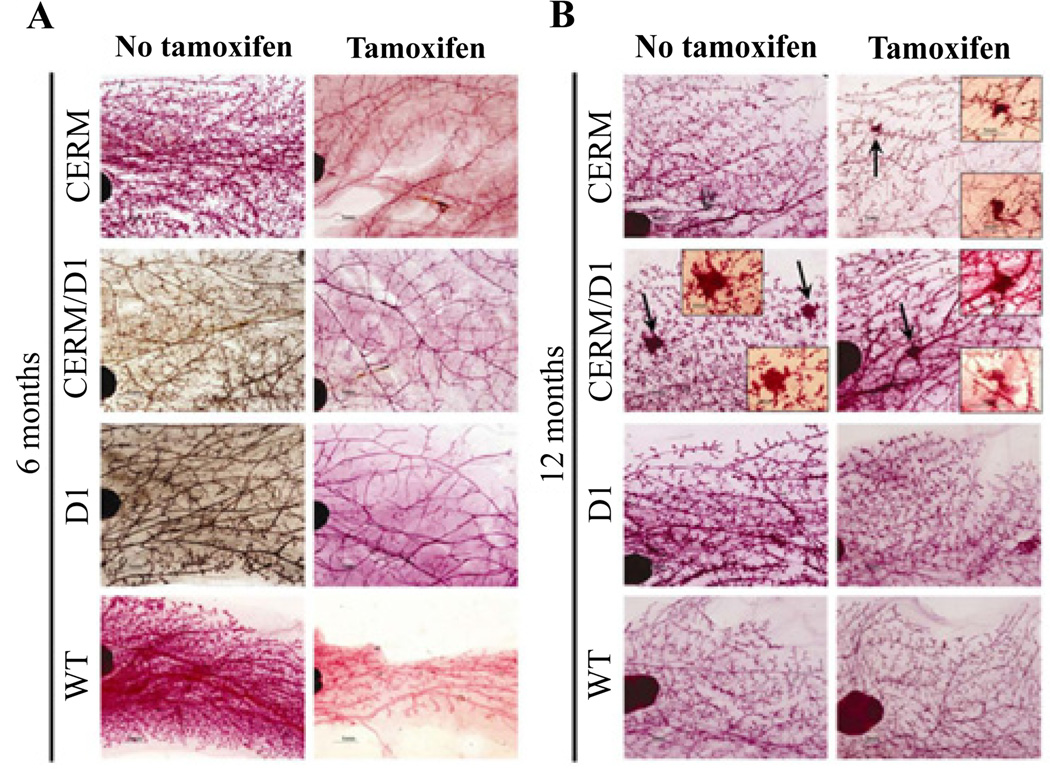
Representative mammary gland whole mounts without and with tamoxifen treatment in six-month and one-year old CERM, CERM/D1, D1 and WT mice Representative whole mount images of six-month and one-year old CERM, CERM/D1, D1 and WT mice treated with tamoxifen for two months demonstrating uniform ductal regression in all genotypes when treated at four months of age and presence of multiple HANs in CERM and CERM/D1 mice when treated at ten months of age (arrows and insets).

To test if resistance to tamoxifen-induced ductal regression was an acquired phenotype over time in the different models and not a direct result of ERα or cyclin D1 expression alone or in combination, the response to tamoxifen was also determined in four-month-old mice and compared to the results from the ten-month-old mice. WT control mice showed expected ductal regression at six months of age, two months after initiation of tamoxifen treatment. Similar patterns of ductal regression were seen in six-month-old CERM, CERM/D1 and D1 mice (Figure 2). HANs were found following tamoxifen treatment only in the CERM, CERM/D1 and D1 mice when the intervention was initiated at age ten months (Figures 1 and 2).

### Mammary adenocarcinomas expressed similar expression levels of hormone receptors, cyclin D1, Ki67 and ErbB2 irrespective of genotype or treatment group

**Figure 3:**
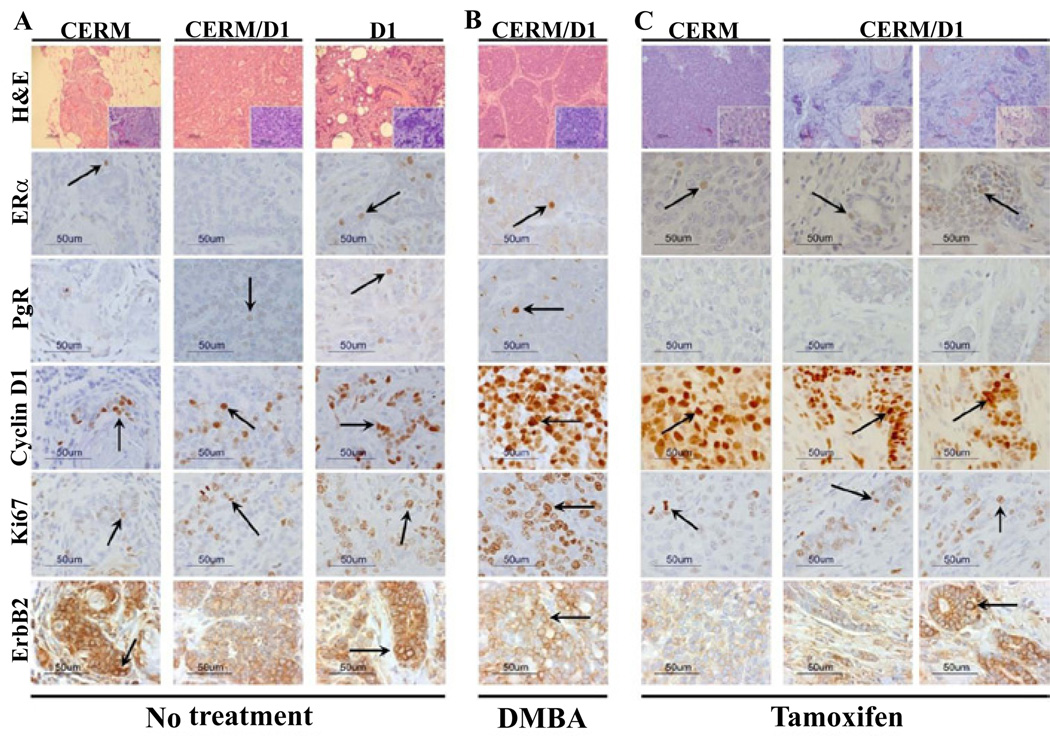
Invasive adenocarcinomas demonstrated expression of hormone receptors and cyclin D1 Representative panels illustrating H&E staining and immunohistochemistry for ERα, PgR, cyclin D1, Ki67 and ErbB2 in mammary adenocarcinomas from CERM, CERM/D1 and D1 mice. Arrows indicate representative stained cells with either nuclear (ERα, PgR, cyclin D1, Ki67) or higher intensity 2+ membrane (ErbB2) staining. Size markers are indicated on all panels.

Immunohistochemistry was used to compare relative expression levels of the hormone receptors ERα and PgR, cyclin D1, Ki67 and ErbB2 in the mammary adenocarcinomas that developed in the different genotypes across treatment groups. Hormone receptor, cyclin D1, Ki67 and ErbB2 expression were detected in all the mammary adenocarcinomas without any significant differences between genotypes or treatment groups (Figure 3).

## Discussion

The most significant finding of the study was the persistence of hyperplasia and development of adenocarcinomas on tamoxifen treatment when it was initiated at ten months of age. These findings are consistent with the appearance of tamoxifen resistance in these mice. It is possible that the cancers and persistent hyperplasias demonstrated preexisting molecular profiles pre-disposing them to tamoxifen resistance as has been suggested for women receiving tamoxifen treatment [40]. Consistent with this, primary prevention with tamoxifen in women was unable to reduce the incidence of ERα positive breast cancers developing on tamoxifen although it did decrease the incidence of ERα positive breast cancers post-treatment [41]. One caveat of this mouse study was that the tamoxifen intervention was relatively short (two months) whereas in women the current recommended duration is between two and five years. It is possible that a longer duration tamoxifen treatment would have been associated with a reduction in hyperplasia prevalence. However, this is balanced against the observation that cancer developed on tamoxifen in the CERM and CERM/D1 genotypes and this was associated with an increase in the percentage of mice with multiple hyperplastic foci in the CERM mice, results that are compatible with the notion that tamoxifen resistance is present in a subset of mammary epithelial cells in these mice by ten months of age.

Timing of interventions to prevent breast cancer may be critical. As discussed above primary prevention with tamoxifen in women was more effective post-treatment than during treatment [41]. In the study here tamoxifen administered at four months of age uniformly induced ductal regression and no resistant hyperplasias or cancers appeared. The different results following tamoxifen treatment at four and ten months of age suggests that time-dependent changes in the mammary epithelial cells following expression of the ERα and D1 transgenes may be required for the appearance of tamoxifen resistance. Time-dependent alterations in salivary epithelial cells following expression of an oncoprotein are known to be responsible for the differences in regression of hyperplasia following down-regulation of the initiating oncoprotein [42,43]. It is possible the similar molecular time-dependent changes occur in the mammary epithelium following ERα and D1 over-expression that can interrupt the response to tamoxifen.

In women, the majority of tamoxifen resistant breast cancers express ERα [44] with co-expression of cyclin D1 with ERα frequently found in cancers resistant to tamoxifen [22,24–28]. Down-regulation of cyclin D1 is associated with tamoxifen induced growth inhibition [45,46]. Cyclin D1 has been shown to enhance the growth response to estrogen and progesterone [21]. The adenocarcinomas that developed both on and off tamoxifen in this study demonstrated similar patterns of gene expression with both detectable hormone receptor expression, principally ERα, and cyclin D1 expression.

The genetically engineered transgenic models studied here demonstrate significant parallels with human breast cancer development both in the initiating pathways and the resultant pathophysiology. In women, estimates of DCIS progression to invasive cancer range between 14 and 50% [47]. Similarly, in the CERM model, whereas ~25% of the mice demonstrate hyperplasia at age one year, invasive cancer is found in less than five percent of the mice at the same timepoint. The higher prevalence of hyperplasia and DCIS as compared to invasive cancer in the CERM mice models the higher prevalence of benign breast disease as compared to invasive cancer in women [48]. Possible next steps in studies utilizing these genetically engineered mouse models would be to directly test if earlier intervention with tamoxifen could prevent emergence of tamoxifen-resistant hyperplasias and cancers as well as to investigate if adding a therapeutic agent such as a CDK4/6 inhibitor targeting the cyclin D1 signaling pathway [49] might limit development of tamoxifen resistance.

## Abbreviations

CERM: Conditional ERα in Mammary tissue
ERα: Estrogen Receptor α
PgR: Progesterone Receptor
HAN: Hyperplastic Alveolar Nodule
DMBA: 7, 12-dimethylbenz [a] anthracene
DCIS: Ductal Carcinoma In Situ
